# Discrepant expressive language lateralization in children and adolescents with epilepsy

**DOI:** 10.1002/acn3.51594

**Published:** 2022-08-24

**Authors:** Alisa Pasichnik, Melissa Tsuboyama, Ali Jannati, Clemente Vega, Harper L. Kaye, Ugur Damar, Jeffrey Bolton, Scellig S. D. Stone, Joseph R. Madsen, Ralph O. Suarez, Alexander Rotenberg

**Affiliations:** ^1^ Neuromodulation Program and Division of Epilepsy and Clinical Neurophysiology, Department of Neurology Boston Children's Hospital, Harvard Medical School Boston Massachusetts USA; ^2^ F. M. Kirby Neurobiology Center Boston Children's Hospital, Harvard Medical School Boston Massachusetts USA; ^3^ Berenson‐Allen Center for Noninvasive Brain Stimulation and Division of Cognitive Neurology, Department of Neurology Beth Israel Deaconess Medical Center, Harvard Medical School Boston Massachusetts USA; ^4^ Neuropsychology Center, Department of Psychiatry Boston Children's Hospital, Harvard Medical School Boston Massachusetts USA; ^5^ Behavioral Neuroscience Program Boston University School of Medicine Boston Massachusetts USA; ^6^ Department of Neurosurgery Boston Children's Hospital, Harvard Medical School Boston Massachusetts USA; ^7^ Department of Radiology Boston Children's Hospital, Harvard Medical School Boston Massachusetts USA

## Abstract

Neuronavigated transcranial magnetic stimulation (nTMS) has emerged as a presurgical language mapping tool distinct from the widely used functional magnetic resonance imaging (fMRI). We report fMRI and nTMS language‐mapping results in 19 pediatric‐epilepsy patients and compare those to definitive testing by electrical cortical stimulation, Wada test, and/or neuropsychological testing. Most discordant results occurred when fMRI found right‐hemispheric language. In those cases, when nTMS showed left‐hemispheric or bilateral language representation, left‐hemispheric language was confirmed by definitive testing. Therefore, we propose nTMS should be considered for pediatric presurgical language‐mapping when fMRI shows right‐hemispheric language, with nTMS results superseding fMRI results in those scenarios.

## Introduction

Surgical resection of a seizure focus is often the only curative option for focal epilepsy. Language mapping is needed to guide surgical planning when the seizure focus may be in proximity of expressive or receptive language areas, particularly in the dominant hemisphere.^1^, ^2^ Conventional methods for language lateralization and/or localization such as electrocortical stimulation (ECS)^3^, ^4^, ^5^ and the intracarotid amobarbital procedure, that is, the Wada test,^6^, ^7^, ^8^ are widely accepted as gold standard for lateralizing cortical areas involved in expressive and receptive language functions prior to resective neurosurgery.^9^ However, the need for patient cooperativity limits the use of these techniques in the pediatric population.^8^, ^10^ Functional magnetic resonance imaging (fMRI) is a noninvasive language‐mapping technique that relies on the blood‐oxygen‐level‐dependent (BOLD) signal.^11^, ^12^, ^13^, ^14^, ^15^ However, fMRI requires patients to remain stationary in an MRI scanner for the duration of testing. This poses a problem for younger pediatric patients, and for those with anxiety, dysregulated behavior, or developmental delay. The option for sedation during fMRI adds a confounding variable to interpretation of test results, as fMRI relies upon detecting the *activation* of functionally relevant cortex.

Neuronavigated transcranial magnetic stimulation (nTMS) offers an alternative to fMRI as a more flexible noninvasive functional mapping method. nTMS relies on focal cortical stimulation through induction of an electric current and disruption of an ongoing language task. In contrast to fMRI, nTMS language mapping relies upon temporary *de‐activation*, or disruption of activity, in functionally relevant cortex.

Although fMRI has become a common noninvasive method for functional presurgical mapping, functional localization by fMRI, and by ECS have at times produced discordant results.^5^ In previous studies, nTMS has been shown to have increased sensitivity for language lateralization, while fMRI has an increased risk for false negative findings, both when compared to direct cortical stimulation (DCS).^16^ Additionally, hemispheric dominance by nTMS correlates with increased risk of surgically related language impairment if surgery is done within the same hemisphere.^17^ However, these studies were performed in adult populations, and we now look at language lateralization discrepancies in the pediatric population.

Given the potential nTMS utility in presurgical mapping, and wide use of fMRI, we explore the occurrences of language localization discrepancy between the two modalities. Specifically, we (1) explore the circumstances under which the two techniques produce discordant results for the lateralization of expressive language; and (2) compare fMRI and nTMS results to “gold standard” techniques in a cohort of pediatric patients evaluated for epilepsy surgery.

## Methods

### Participants

As part of the Epilepsy Program at Boston Children's Hospital, patients underwent fMRI and nTMS presurgical language mapping for epilepsy surgery evaluation from 2015 to 2019. Only those with a unilateral seizure focus, who successfully underwent language mapping via both nTMS and non‐sedated fMRI and confirmatory testing, that is, ECS, Wada, or pre‐ and postsurgical neuropsychological testing, were included. Additional demographic and history information were obtained from medical records, as approved by the Boston Children's Hospital's Institutional Review Board (IRB‐P00020115).

### nTMS for functional mapping of expressive language

Each MRI was converted to a three‐dimensional head surface and reconstructed brain using Nexstim 4.3/5.1 software (Nexstim, Finland). Surface electromyography (EMG) electrodes were placed on bilateral abductor pollicis brevis (APB) muscles with the ground electrode placed on the underside of the right forearm. Single pulses were applied to the APB region of the motor cortices, using frameless stereotaxy and a figure‐of‐eight coil. Resting motor threshold (rMT), defined as the minimum intensity needed to elicit an APB motor evoked potential ≥50 *μ*V on ≥50% of trials, was obtained. During object‐naming task, a series of black and white images were presented at eye‐level for 1 sec with a 2‐sec inter‐picture interval. Only objects named correctly during baseline testing were presented during mapping. Language mapping was performed at 90–110% rMT, and stimulation was delivered in 1‐sec 5 Hz trains to pars triangularis, approximating Broca's region and its homolog in the two hemispheres. For patients who experienced pain due to stimulation intensity/frequency, parameters were adjusted to minimize discomfort.

### nTMS analysis

Responses during the mapping protocol were manually reviewed and scored as correct, no response (complete speech arrest), or semantic error (paraphasic error). Exclusive presence of either error type in only one hemisphere was interpreted as lateralized expressive language in that hemisphere. Presence of either error type in both hemispheres was interpreted as bilateral expressive language representation and hemispheric dominance was not quantified in these cases. These errors were then categorized as resulting from left‐ or right‐hemisphere stimulation. Once mapping of both hemispheres was complete, patients were recorded as having left, right, or bilateral expressive language.

### Structural imaging for fMRI and nTMS

MR imaging was obtained using a 3 T MRI scanner (Siemens, Berlin, Germany) configured with a 64‐channel head coil. All patients received a 3D sagittal T1‐weighted magnetization prepared rapid acquisition gradient echo (MPRAGE), TR/TE = 2530/3.39 msec, flip angle = 7^o^, 24 cm FOV, 256 × 256 matrix, 1.0 mm slices.

### Behavioral fMRI tasks

fMRI was acquired using a 64‐channel head coil with typical scanning parameters consisting of: TR/TE = 2500/31 msec; flip angle = 90^o^, 24 cm FOV; 128 × 128 matrix, 3.0–4.0 mm slices. Language tasks were selected to match level of anticipated patient participation, ranging from overt articulation of response words (antonym‐generation, verb‐generation, or object‐naming), to overt button presses (auditory descriptive decision task). The auditory descriptive decision tasks were completed in 5 min with each run lasting 30 sec. Two different versions were run, one with rest and one with backwards speech, and then a control condition. For each scan, there were five runs of active task and five runs of control task, which is 2.5 min of task condition per scan. The expressive language tasks were run twice with backward speech and twice with rest. Visual and auditory stimuli were delivered using LCD and earphones during MRI scanning (NordicNeuroLabs, Bergen, Norway).

### fMRI analysis

Standard preprocessing of fMRI volumes was done including low bandpass filtering, spatial smoothing with 8 mm kernel, and rigid alignment of all BOLD time‐series acquisition volumes to their mean. Activated cortical regions were then identified by Statistical Parametric Mapping^18^ (Wellcome Laboratories, UK) based on the correlation between BOLD signal and stimulus presentation. We generated BOLD contrasts for language task compared to control baseline conditions (rest and backward speech). Language dominance was determined by statistical parametric mapping (using SPM) based on the correlation between blood‐oxygen‐level‐dependent (BOLD) signal fluctuations and the stimulus presentation paradigm. Activation maxima in fMRI maps were identified based on uncorrected *P* ≤ 0.01 to avoid excessive family‐wise errors. fMRI activation maps were overlaid onto T1‐weighted MRIs. Each expressive language map was scored as left, right, or bilateral. Each language‐based fMRI task was analyzed separately, and in all cases the language lateralization per task was concordant.

### Comparison to gold standard results

In cases where names and fMRI results were discordant, results were compared to ECS, Wada test, or postsurgical naming decline as measured by the Boston Naming Test (BNT).^19^ The BNT is a standard measure of object‐naming, which is similar to the naming tasks used for nTMS and fMRI language testing. When ECS for expressive language mapping was performed, the amount of inferior frontal gyrus coverage (either by grids or stereotactic EEG) varied based on the preimplantation hypothesis as to the location of the seizure onset zone. Thus, the areas stimulated by ECS did not always directly overlap with the entirety of the area covered by nTMS but was sufficient to determine language lateralization. Visual object‐naming tasks similar to those used during fMRI were implemented during ECS testing. Wada testing for language lateralization consisted of visual object‐naming tasks, repetition, sequencing tasks, and following commands (for comprehension).

## Results

Of the 19 consecutive patients (ages 9–22) who completed both fMRI and nTMS mapping, concordant results were identified in 11. Epilepsy etiologies included tuberous sclerosis, focal cortical dysplasia, stroke, tumor, mesial temporal sclerosis, and traumatic brain injury.

**Figure 1:**
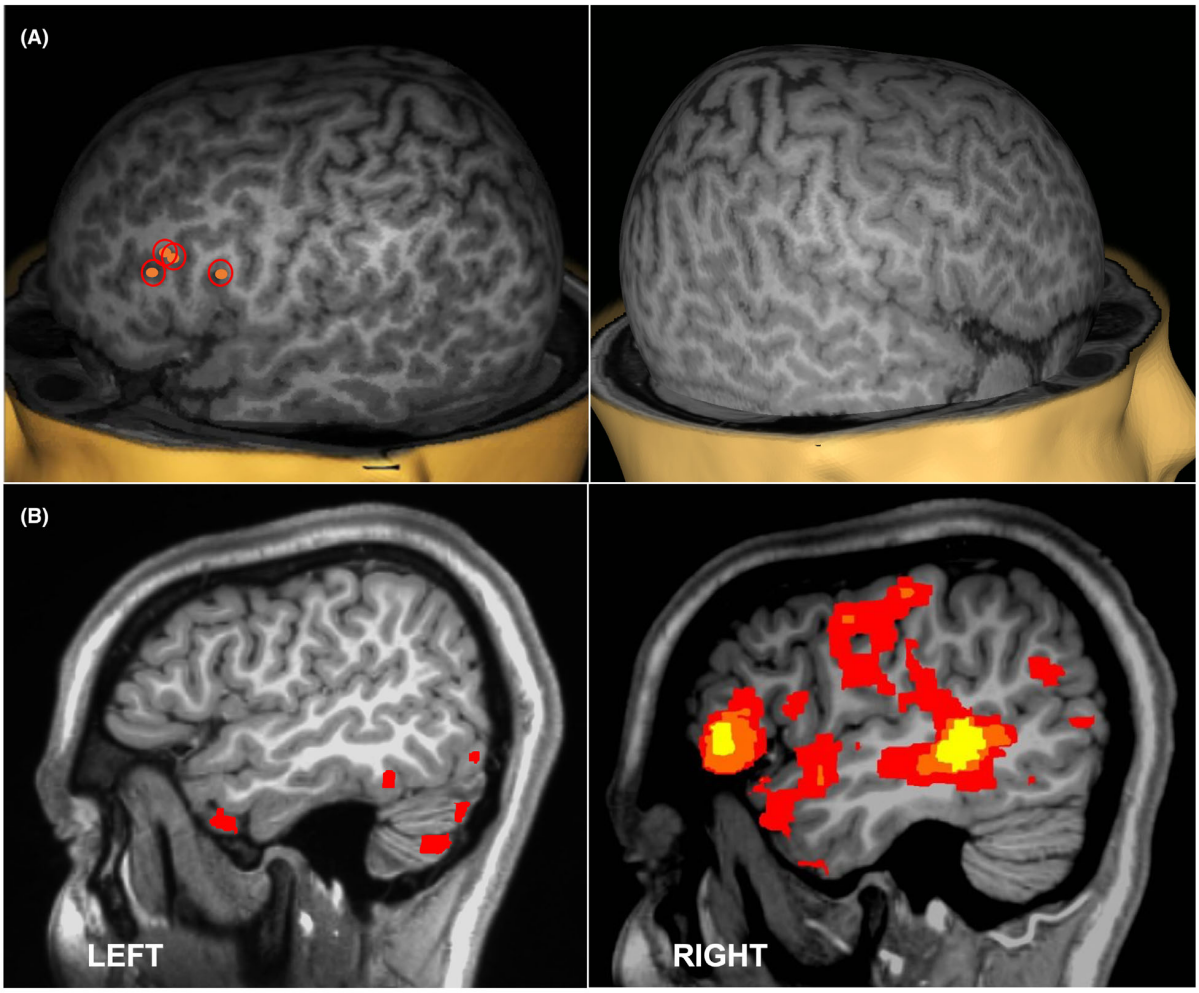
Language maps in a right handed patient with a left‐hemispheric focal cortical dysplasia. (A) nTMS‐induced language errors, where the orange dots circled in red indicate points of stimulation that induced expressive language errors in the *left hemisphere only*; (B) fMRI activation for expressive language depicting *right hemisphere representation only*. nTMS findings were supported by electrocortical stimulation also finding left hemisphere language localization. [Colour figure can be viewed at wileyonlinelibrary.com]

Of the six instances where fMRI reported right‐hemispheric language, *n* = 5 (83%) were discordant with nTMS (Fig. 1). Of the eight instances where fMRI reported bilateral language, *n* = 3 (38%) were discordant with nTMS findings, all of which were found strictly left‐hemispheric language. In all instances in which fMRI reported left‐hemispheric language, fMRI and nTMS findings were concordant. Table 1 provides a summary of fMRI versus nTMS language lateralization results, and Table 2 compares fMRI and nTMS results to confirmatory tests. Among the discordant group (*n* = 8), ECS corroborated nTMS findings in the two subjects who demonstrated right‐hemispheric language by fMRI but left‐hemispheric language by nTMS. Of the three subjects with right‐hemispheric language by fMRI but bilateral language by nTMS, presence of left‐hemispheric language was confirmed by ECS in *n* = 2; no confirmatory testing was completed for the third subject.

**Table 1:** Discrepancy in language localization between fMRI and nTMS measures.

	TMS left	TMS bilateral	TMS right	Total # subjects
fMRI Left	5	0	0	5
fMRI bilateral	**3**	5	0	8
fMRI right	**2**	**3**	1	6
Total # subjects	10	8	1	19

Values in bold correspond to instances of fMRI‐TMS discrepancy; Chi‐Sq *P* < 0.05.

**Table 2:** Per subject demographic data, clinical data, and language mapping results using fMRI versus nTMS versus confirmatory tests.

Subject	Epileptic hemisphere	Grid or sEEG Coverage	Age at Testing (years)	Sex	Etiology	Location of SOZ	fMRI	nTMS	Confirmatory testing		
									Wada	ECS	BNT score
1	Left	Frontal, parietal & temporal	9	F	Vasculitis	Temporal & occipital	Right	Left		Left	Decline
2	Left	Frontal & temporal	11	M	Malformation of cortical development	Frontal	Right	Left		Left	
3	Left	N/A	10	F	Focal cortical dysplasia	Frontal	Right	Bilateral			
4	Right	Frontal & parietal	22	F	Traumatic brain injury	Frontal	Right	Bilateral		Right	No decline
5	Left	Frontal, parietal & temporal	15	F	Perinatal stroke	Frontal & parietal	Right	Bilateral		Left	
6	Left	Frontal, parietal, temporal, & occipital	13	F	Inflammatory (Rasmussen's encephalitis)	Temporal & insular	Bilateral	Left	Left	Not tested	Decline
7*	Left	Frontal, parietal & temporal	16	M	Focal cortical dysplasia (based on MRI)	Frontal	Bilateral	Left		Left	
8	Left	Frontal & temporal	11	F	Mesial temporal sclerosis + gliosis	Temporal	Bilateral	Left		Not tested	Decline

sEEG, stereotactic EEG; SOZ, seizure onset zone; ECS, electrocortical stimulation (using grids or sEEG); BNT, Boston Naming Test, comparing postsurgical scores to presurgical scores.

* Subject 7 did not undergo resective surgery.

## Discussion

Presurgical language mapping findings confirmed by converging evidence from multiple modalities increases the confidence of decision‐making in children undergoing epilepsy surgery evaluation. In a previous study it was found that multi‐modal approaches to language localization using ECS, TMS and fMRI increased the sensitivity of ECS by finding more true positive areas of language localization.^20^ In contrast, we explored instances of discordance between fMRI and nTMS to determine which test was more reliable in those situations using ECS, Wada and changes in BNT score as the gold standard. In a study comparing Wada to ECS, left language dominance by Wada was concordant with ECS data in all cases (*n* = 15).^21^ However, when Wada demonstrated right‐hemispheric or bilateral language representation, ECS data were discordant in seven of 19 cases.^21^ Thus, confirmatory testing by ECS is necessary in those instances. The majority of discordance between fMRI and nTMS results occurred when fMRI indicated right‐hemispheric language whereas nTMS identified either left hemisphere or bilateral representation. In those cases, the involvement of the left hemisphere was confirmed by ECS, Wada, or BNT. Patients with atypical language representation were not specifically selected for but were highly represented in our study due to these patients having an increased likelihood of undergoing additional confirmatory language testing (i.e., TMS).

Discordant results in which fMRI indicates right‐hemispheric language while nTMS demonstrates left or bilateral hemispheric language suggests that at a minimum left‐hemispheric language is likely to be present. Right‐hemispheric language is a rare occurrence^22^, ^23^, ^24^, ^25^ such that when it is demonstrated on fMRI, nTMS should be performed to validate these findings. Thus, performing nTMS for language lateralization is strongly recommended in all children undergoing epilepsy surgery in close proximity to eloquent language cortex, especially when fMRI does not show the expected left‐hemispheric language lateralization. These findings correlate with previous studies that have found TMS to have a higher sensitivity of finding left‐ hemispheric language.^26^ Additionally, these findings complement adult studies that have found increased sensitivity for language lateralization with nTMS compared to fMRI as well as correlation of nTMS lateralization with risk of postsurgical deficits in the same hemisphere.^16^, ^17^

Although the reason for discrepancies in language lateralization between fMRI and nTMS modalities is unclear, one cause for such discrepancies may be the difference in the nature of the detected signal. Positive results in nTMS language mapping represent external stimulations that directly disrupt the cortical activity that is necessary for expressive language, whereas fMRI results represent a correlation between changes in regional blood flow that are associated with participation in an expressive language task. Thus, the BOLD results detected by fMRI language mapping in some patients may not be as specific to the brain regions that are critical for expressive language as nTMS. Additional investigations of the BOLD response in subjects with discordant data may provide insight into mechanistic limitations of functional mapping via fMRI, thus guiding physicians on which patients with epilepsy undergoing presurgical functional mapping are better suited for fMRI versus nTMS. As it stands, nTMS results provide evidence complementary to those obtained by fMRI that can be critical for determining the hemispheric lateralization of expressive language in patients under consideration for epilepsy surgery.

## Conflict of Interest

A.R. is a founder and advisor for Neuromotion and PrevEp, and serves on the scientific advisory board or has consulted for Cavion, Epihunter, Gamify, Neural Dynamics, NeuroRex, Praxis, Roche, Otsuka, and is listed as an inventor on a patent related to integration of TMS and EEG. The remaining authors declare that the research was conducted in the absence of any commercial or financial relationships that could be construed as a potential conflict of interest.
